# Therapeutic effects of Poncirus fructus on colonic dysfunction and visceral pain in a zymosan-induced irritable bowel syndrome mouse model through interstitial cells of Cajal and ion channel modulation

**DOI:** 10.3389/fphar.2025.1639592

**Published:** 2025-10-15

**Authors:** Na Ri Choi, Seok-Jae Ko, Woo-Gyun Choi, Daehwa Jung, Sang Chan Kim, Dong Wook Lim, Yun Tai Kim, Joo Han Woo, Jae-Woo Park, Byung Joo Kim

**Affiliations:** ^1^ Department of Longevity and Biofunctional Medicine, Pusan National University School of Korean Medicine, Yangsan, Republic of Korea; ^2^ Department of Digestive Diseases, College of Korean Medicine, Kyung Hee University, Seoul, Republic of Korea; ^3^ Department of Korean Internal Medicine, Kyung Hee University College of Korean Medicine, Kyung Hee University Hospital at Gangdong, Seoul, Republic of Korea; ^4^ Department of Pharmaceutical Engineering, Daegu Hanny University, Gyeongsan, Republic of Korea; ^5^ College of Oriental Medicine, Daegu Haany University, Gyeongsan, Republic of Korea; ^6^ Division of Food Functionality, Korea Food Research Institute, Wanju-gun, Republic of Korea; ^7^ Department of Food Biotechnology, Korea University of Science and Technology, Daejeon, Republic of Korea; ^8^ Department of Physiology, College of Medicine, Dongguk University, Gyeongju, Republic of Korea

**Keywords:** irritable bowel syndrome, Poncirus fructus, zymosan-induced, colitis, electrophysiology, gastrointestinal motility

## Abstract

**Purpose:**

Irritable bowel syndrome (IBS) is a prevalent gastrointestinal disorder with few effective long-term treatments. This study investigated the therapeutic potential of *Poncirus fructus* extract (PFE) for IBS-like symptoms, focusing on interstitial cells of Cajal (ICCs), visceral pain–related ion channels, inflammation, and gut microbiota.

**Methods:**

A zymosan-induced colitis mouse model was employed to mimic IBS-associated inflammation and pain. Electrophysiological recordings were performed in murine colonic ICCs and HEK293T cells overexpressing TRPV1, TRPV4, TRPA1, NaV1.5, or NaV1.7 channels. Additional analyses included histology, TNF-α measurement, behavioral pain assessments, and gut microbiota profiling.

**Results:**

PFE depolarized ICC pacemaker potentials in a dose-dependent manner through HCN channel activation and M3 muscarinic receptor–mediated PLC-PKC signaling involving p38 MAPK and JNK pathways. *In vivo*, PFE improved colon length, reduced tissue inflammation and damage, lowered TNF-α levels, and alleviated pain-related behaviors in zymosan-treated mice. Gut microbiota analysis revealed increased abundance of Lachnospiraceae following PFE treatment. Electrophysiology showed that PFE inhibited TRPV1 and NaV1.5/1.7 currents, enhanced TRPV4 current, and had no effect on TRPA1 current.

**Conclusion:**

PFE exerts multi-target effects by modulating ICC activity, suppressing inflammation, and regulating key ion channels involved in visceral pain. These findings suggest that PFE has therapeutic potential for the management of IBS symptoms.

## 1 Introduction

Irritable bowel syndrome (IBS) is a prevalent functional gastrointestinal (GI) disorder marked by changes in bowel habits, abdominal discomfort, and increased visceral sensitivity (Marasco et al., 2025; Nozu et al., 2025). Despite its high prevalence, effective and safe long-term treatments remain limited, highlighting the need for alternative therapeutic strategies (Zhang et al., 2024; Jeong et al., 2025). In recent years, botanical medicines have gained considerable interest due to their diverse bioactivities and long history of traditional use.

Interstitial cells of Cajal (ICCs) act as pacemaker cells within the GI tract, generating rhythmic electrical slow waves that regulate smooth muscle contractions and coordinate intestinal motility (Lee J et al., 2024; Baker et al., 2025). Dysfunction of ICCs has been associated with impaired gut motility and visceral pain, both of which are key features of IBS (Foong et al., 2020; Patejdl, 2024).

The zymosan-induced colitis model is commonly used to mimic IBS-related GI inflammation and pain (Choi et al., 2023; Choi et al., 2024a; Choi et al., 2024b; Siting et al., 2025). Zymosan, a yeast-derived substance, triggers an inflammatory response in the colon that resembles the pathophysiology observed in IBS patients and this model serves as a valuable platform for evaluating potential therapies targeting inflammation, motility disturbances, and pain hypersensitivity associated with IBS (Choi et al., 2023; Choi et al., 2024a; Choi et al., 2024b; Siting et al., 2025). Many current therapeutic approaches for IBS focus on modulating ion channels involved in pain perception and inflammation (Saito et al., 2009; Verstraelen et al., 2015; Fuentes and Christianson, 2016; Beckers et al., 2017; Alaimo and Rubert, 2019; Choi et al., 2023; Choi et al., 2024a; Choi et al., 2024b). Several transient receptor potential (TRP) channels, such as TRPV1, TRPV4, and TRPA1, have been identified as key mediators of visceral pain signaling in the gut (Beckers et al., 2017; Alaimo and Rubert, 2019). In parallel, voltage-gated sodium (NaV) channels, particularly NaV1.5 and NaV1.7, play critical roles in GI function and nociceptive transmission (Verstraelen et al., 2015; Choi et al., 2023; Choi et al., 2024a; Choi et al., 2024b). Consequently, these channels offer valuable targets for creating new therapeutic approaches to IBS.

Poncirus fructus (PF), or *Poncirus trifoliata* (L.) Raf. from the Rutaceae family, has a long history of use in traditional medicine for managing GI ailments (Jang et al., 2018; Lee K et al., 2024). Recent studies suggest that PF possesses anti-inflammatory and analgesic properties (Shin et al., 2006; Khan et al., 2019). However, its precise mechanisms of action in the context of IBS have yet to be fully elucidated.

In this study, we investigated the effects of Poncirus fructus extract (PFE) on IBS-like symptoms using a zymosan-induced colitis model. Our focus was on assessing PFE’s impact on ICCs function, colonic inflammation, pain-associated behaviors, and its modulatory effects on key ion channels involved in pain sensation, including TRP and NaV channels. Through electrophysiological recordings, histological evaluations, behavioral analyses, and gut microbiota profiling, this research aims to elucidate the therapeutic potential of PFE for alleviating visceral pain and GI dysfunction in IBS.

## 2 Methods

### 2.1 Sample preparation

PF was purchased from Korea Natural Product Central Bank (https://kobis.re.kr/npcb/uss/main.do; Ochang, Republic of Korea) and extracted using a 95% ethanol solution under reflux at 45 °C for 72 h. The PFE was concentrated using a rotary evaporator and subsequently freeze-dried. The final yield of the dried extract was 12.9%.

### 2.2 Quantification of flavonoids from PFE

PFE and standard metabolites, including naringin and poncirin, were dissolved in 50% methanol, filtered through a 0.45 µm PVDF syringe filter, and used for analysis. HPLC was performed using a system equipped with a UV detector set at 280 nm. A 10 μL volume of each sample was injected into a C18 column (4.6 × 250 mm, 5 μm). The HPLC conditions used for quantification of NA and PO in the PFE are summarized in Table 1. The mobile phase consisted of 0.1% formic acid in water (solvent A) and acetonitrile (solvent B), delivered at a flow rate of 1 mL/min with the column maintained at 30 °C. A gradient elution was applied: solvent A started at 90%, decreased to 60% over 30 min, and returned to 90%, with a total run time of 60 min. All solvents were of HPLC grade.

**TABLE 1:** Condition of HPLC analysis for NA and PO in PFE.

HPLC analysis			
Column	C18 column (4.6 × 150 mm, 5 μm)		
Mobility	Time (min)	(A) 0.1% formic acid	(B) ACN
	0	90	10
	30	60	40
	60	90	10
Flow rate	1 mL/min		
Column temperature	30 °C		
Injection volume	10 μL		
Detection	280 nm		

### 2.3 Preparation of ICCs cultures

For the ICCs experiments, a total of 58 ICR mice, aged between 3 and 6 days, were acquired from Samtako Bio Korea Co., Ltd. (Osan, Republic of Korea). To prepare ICCs cultures, large intestines were harvested from ICR mice aged 3–6 days, including both males and females. The intestines were carefully dissected to remove the mucosal layer, ensuring minimal tissue damage. To facilitate the separation of the smooth muscle layers, the tissues were enzymatically digested using a solution containing collagenase (Worthington Biochemical Corporation, Lakewood, NJ, USA). The enzymatic digestion allowed for the efficient dissociation of cells, which were then suspended and maintained in a smooth muscle growth medium (Clonetics Corp., San Diego, CA, USA). The cultures were incubated at 37 °C in a controlled environment to support cell viability and proliferation. All ICC-related experiments were performed after 12 h of culture to ensure proper cell adaptation and functionality.

### 2.4 Induction of colitis

In the zymosan-induced IBS animal model study, 78 male mice, aged 6–7 weeks and weighing between 23 and 26 g, were utilized. To induce colitis, a 0.1 mL solution of 30 mg/mL zymosan (Sigma-Aldrich, St. Louis, MO, USA), was administered directly into the colon of experimental mice for three consecutive days. This method aimed to trigger an inflammatory response resembling colitis. Following the induction of colitis, the mice were randomly assigned to six experimental groups: (a) naive (n = 13), which did not receive any treatment; (b) control (n = 15), representing the colitis-induced but untreated group; (c) PFE 250 mg/kg (n = 14), a treatment group receiving a low dose of PFE; (d) PFE 500 mg/kg (n = 15), a group receiving a higher dose of PFE; (e) amitriptyline (AMT) (n = 13), an antidepressant commonly prescribed for irritable bowel syndrome (IBS) symptom relief; and (f) sulfasalazine (SSZ) (n = 14), an anti-inflammatory agent widely used in the treatment of GI diseases. AMT and SSZ were included as positive control treatments to compare their effects with PFE in mitigating colitis symptoms (Pruzanski et al., 1997; Iqbal et al., 2025).

### 2.5 Assessment of body weight changes and food intake

To monitor physiological changes associated with colitis induction and treatment, body weight measurements were taken on days 1, 4, 8, and 12. This allowed for the assessment of weight fluctuations over time, providing insight into the potential impact of colitis and therapeutic interventions on overall health. Additionally, total food intake was recorded throughout the experimental period to evaluate dietary consumption patterns. Tracking both body weight and food intake helped determine whether colitis induction or treatment affected metabolic status, appetite, or overall wellbeing in the experimental groups.

### 2.6 Evaluation of colon and stool conditions

To evaluate the impact of zymosan-induced colitis on the colon, both colon weight and length were measured. Colon length was specifically defined as the distance from the cecal end to the anus, allowing for a standardized assessment of potential inflammation-related shortening. Additionally, stool characteristics were analyzed using a structured scoring system. Three independent researchers, blinded to the experimental conditions, assessed stool consistency based on the Bristol Stool Scale. Stool consistency was categorized into four distinct levels: 0 (normal), 1 (moist), 2 (sticky), and 3 (diarrhea). This comprehensive evaluation helped determine the severity of colitis-induced GI changes and treatment effects.

### 2.7 Histological analysis of the colon

For histological analysis, colon tissue samples were first fixed to preserve structural integrity, then embedded in paraffin to facilitate sectioning. Thin tissue sections were prepared and stained using hematoxylin and eosin (H&E) to highlight cellular and morphological features. The stained samples were subsequently examined under a visible-light microscope (Nikon, Tokyo, Japan) to assess histopathological changes, such as inflammation, epithelial damage, and tissue integrity. This analysis provided detailed insights into the extent of colitis-induced tissue alterations and the potential effects of therapeutic interventions.

### 2.8 Quantification of tumor necrosis factor (TNF)-α expression level by RT-qPCR

To quantify the expression level of Tumor Necrosis Factor (TNF)-α, total mRNA was extracted from colonic tissue and the isolated RNA was then reverse-transcribed into complementary DNA (cDNA) using a cDNA synthesis kit containing M-MLV Reverse Transcriptase (Promega, Madison, WI, USA). This process allowed for the conversion of mRNA into cDNA, which could then be used as a template for further quantitative PCR analysis to evaluate the expression levels of TNF-α. The qPCR reaction was run using QuantStudio 1 (Applied Biosystems, Waltham, MA, USA) at the Cardiovascular and Metabolic Disease Core Research Support Center (Busan, Republic of Korea).

### 2.9 Assessment of pain-associated behaviors

Pain-related behaviors were assessed using established methods described in a previous study (Laird et al., 2001). The evaluation focused on specific behavioral indicators, including abdominal licking, full-body stretching, pressing the abdomen against the floor, and arching caused by abdominal contractions. To enhance the reliability of the observations, two independent researchers carefully monitored and recorded these behaviors over a 10-min period. This approach ensured an objective assessment of pain responses associated with colitis induction and treatment effects.

### 2.10 Microbiota analysis

Genomic DNA was extracted from stool samples using the QIAamp PowerFecal DNA Kit (QIAGEN, Hilden, Germany). The V4 region of the bacterial 16S rRNA gene was amplified with unique 8 bp barcodes and sequenced on the Illumina iSeq 100 platform (Nakao et al., 2021). Sequence data were analyzed using the QIIME pipeline, with taxonomic classification performed based on the SILVA 128 reference database (Caporaso et al., 2010; Quast et al., 2013).

### 2.11 Plasmid transfection

For plasmid transfection, HEK293T cells were first cultured in 6-well plates under appropriate growth conditions. The following day, transfection was carried out using a specialized reagent (Thermo Fisher Scientific, Waltham, MA, USA). Plasmid DNA (1.5–2 µg per well) encoding human TRP channels TRPV1, TRPV4, TRPA1, or NaV channels NaV1.5 and NaV1.7 was introduced into the cells. Additionally, pEGFP-N1 was co-transfected to enable visualization of successfully transfected cells.

### 2.12 Electrophysiological experiments

Whole-cell patch-clamp recordings were conducted using an Axopatch 200B amplifier (Molecular Devices, San Jose, CA, USA) to investigate the electrophysiological properties of the cells. Pacemaker potentials in ICCs were assessed in current-clamp mode. To examine TRP channel activity, a holding voltage of −60 mV was applied, with ramp pulses ranging from −100 mV to 100 mV. For NaV channel recordings, membrane currents were measured over a voltage range of −120 mV to 0 mV, with a holding voltage set at −120 mV. The detailed compositions of the internal and external solutions used in these experiments are available in a previously published study (Choi et al., 2023; Lee J et al., 2024).

### 2.13 Statistical analysis

The results are expressed as the mean ± standard error (SE). Variance analysis was conducted using a one-way analysis of variance (ANOVA), followed by Dunnett’s multiple comparison test to compare group differences. All statistical evaluations were carried out using GraphPad Prism 8 software, with statistical significance set at a p-value of less than 0.05.

## 3 Results

### 3.1 Quantification of flavonoids in PFE

**FIGURE 1:**
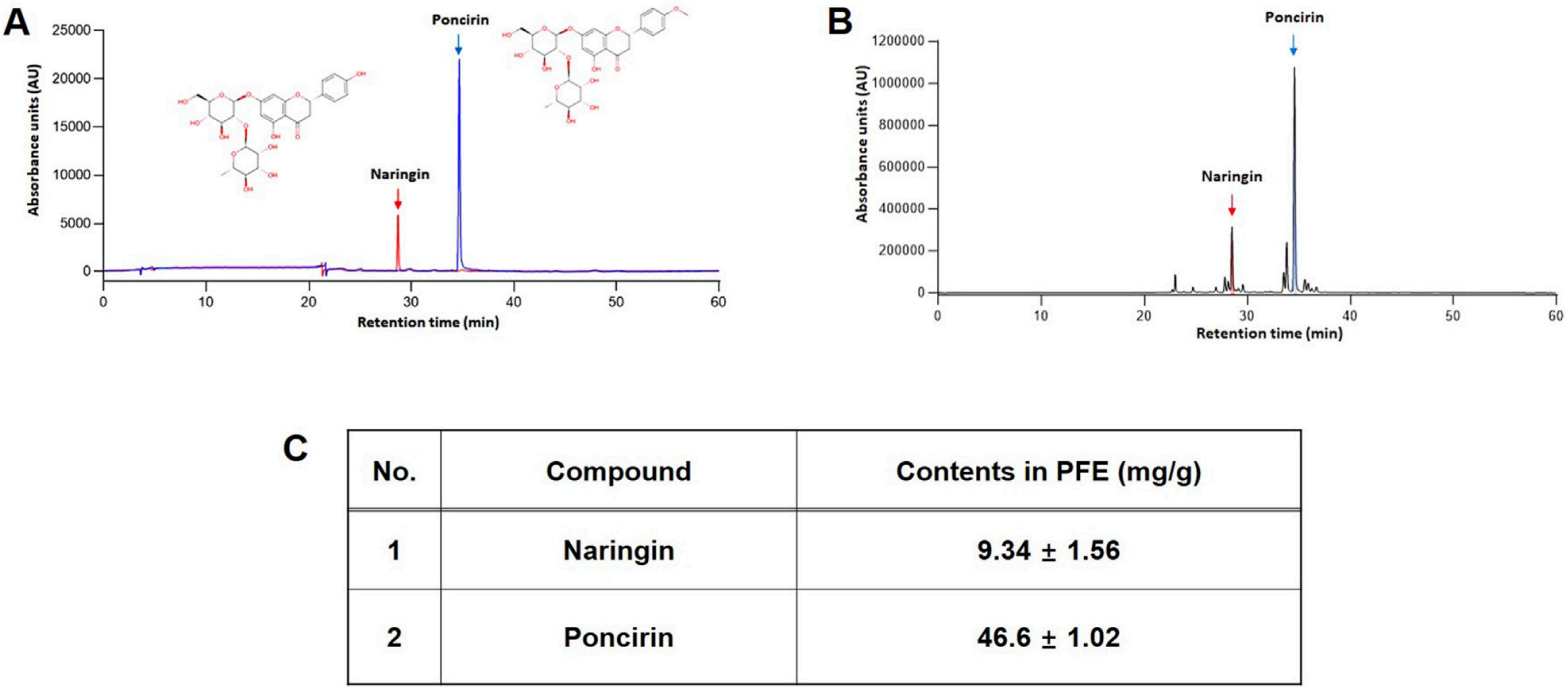
Identification of major metabolites of PFE. HPLC chromatogram of NA, and PO as a standard metabolite’s mixture **(A)** and PFE **(B)**. **(C)** Quantitative analysis in PFE. The concentration of NA and PO were 9.34 ± 1.56 mg/g and 46.6 ± 1.02 mg/g PTE, respectively.

NA and PO are known as the major metabolites of PFE (Lee et al., 2005). Quantitative analysis was performed in triplicate, and the concentrations of NA and PO in PFE were determined to be 9.34 ± 1.56 mg/g and 46.6 ± 1.02 mg/g, respectively. Calibration curves for both metabolites were constructed based on peak area versus concentration, and exhibited excellent linearity (NA, *y = 297,34x-24244*, *R* ^2^ = 0.9995; PO, *y = 227,03x+12,942*, *R* ^2^ = 0.9998). A representative chromatogram of the reference standards and their corresponding peaks in the PFE is shown in Figure 1.

### 3.2 Effects of PFE on the pacemaker potentials of ICCs from murine large intestines

**FIGURE 2:**
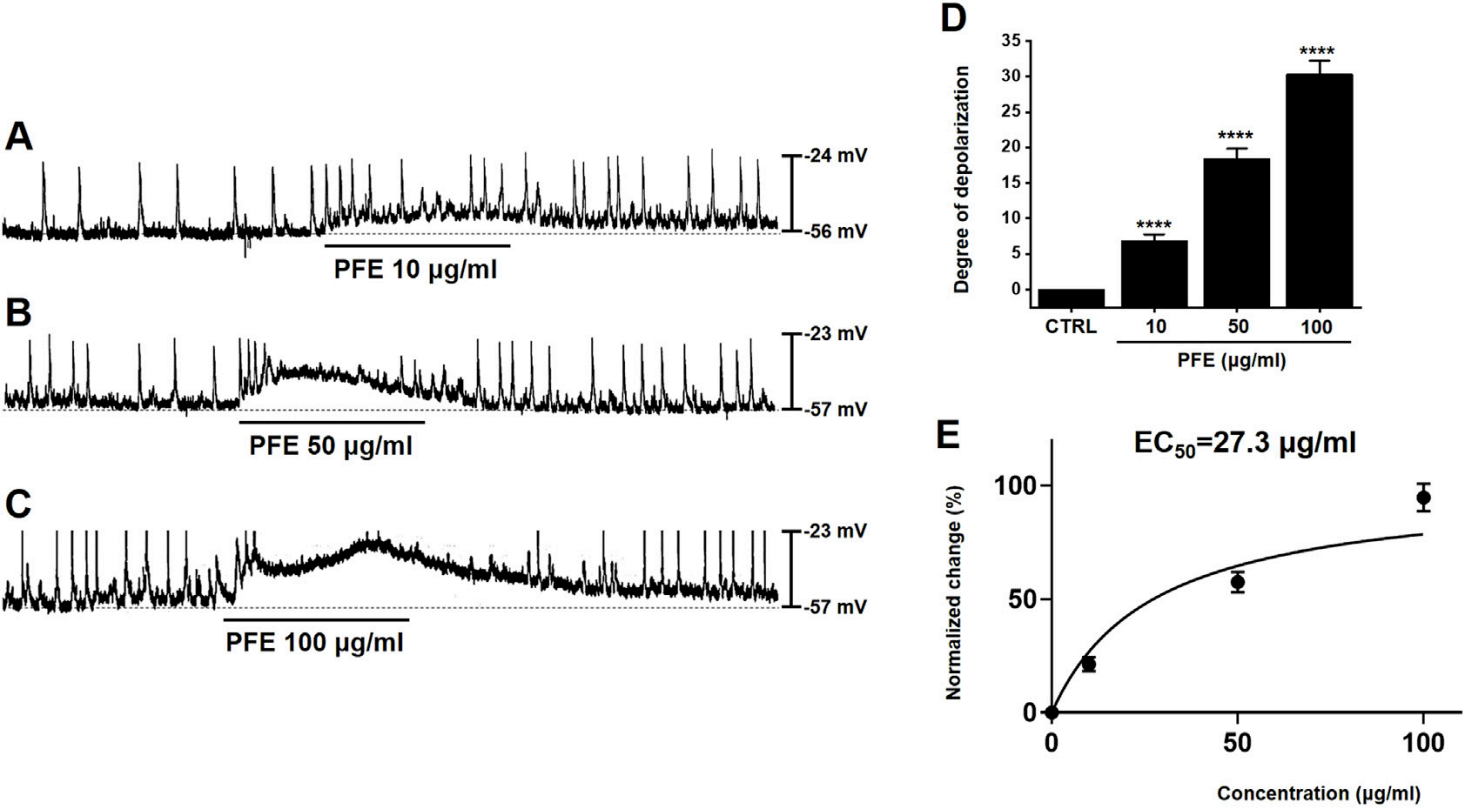
Impact of PFE on pacemaker activity in ICCs from the murine colon. **(A–C)** The administration of PFE led to a dose-dependent depolarization in the pacemaker potential of ICCs. This depolarizing effect was more pronounced at higher concentrations of PFE. **(D)** A summary of the changes in pacemaker potentials following PFE treatment is shown, with the data normalized for comparison. **(E)** EC_50_ is 27.3 μg/mL. Bars indicate mean ± SE. ****p < 0.0001 compared to control. *CTRL: Control.*

We utilized the whole-cell patch-clamp technique to analyze the electrophysiological properties of ICCs in the large intestine. In current-clamp mode (I = 0), ICCs exhibited spontaneous pacemaker potentials, which were depolarized by PFE (10–100 μg/mL) in a concentration-dependent manner (Figures 2A–C). In the presence of PFE, mean degrees of depolarization were 6.8 ± 1.0 mV (n = 7) at 10 μg/mL, 18.4 ± 1.4 mV (n = 8) at 50 μg/mL, and 30.3 ± 1.9 mV (n = 7) at 100 μg/mL (Figure 2D). Based on our analysis, the EC_50_ value for PFE’s depolarizing effect on pacemaker potentials was determined to be 27.3 μg/mL (Figure 2E). These results indicate that PFE modulates ICC activity by depolarizing pacemaker potentials.

### 3.3 Hyperpolarization-activated cyclic Nucleotide-Gate (HCN) channel-mediated modulation of PFE responses in murine large intestinal ICCs

**FIGURE 3:**
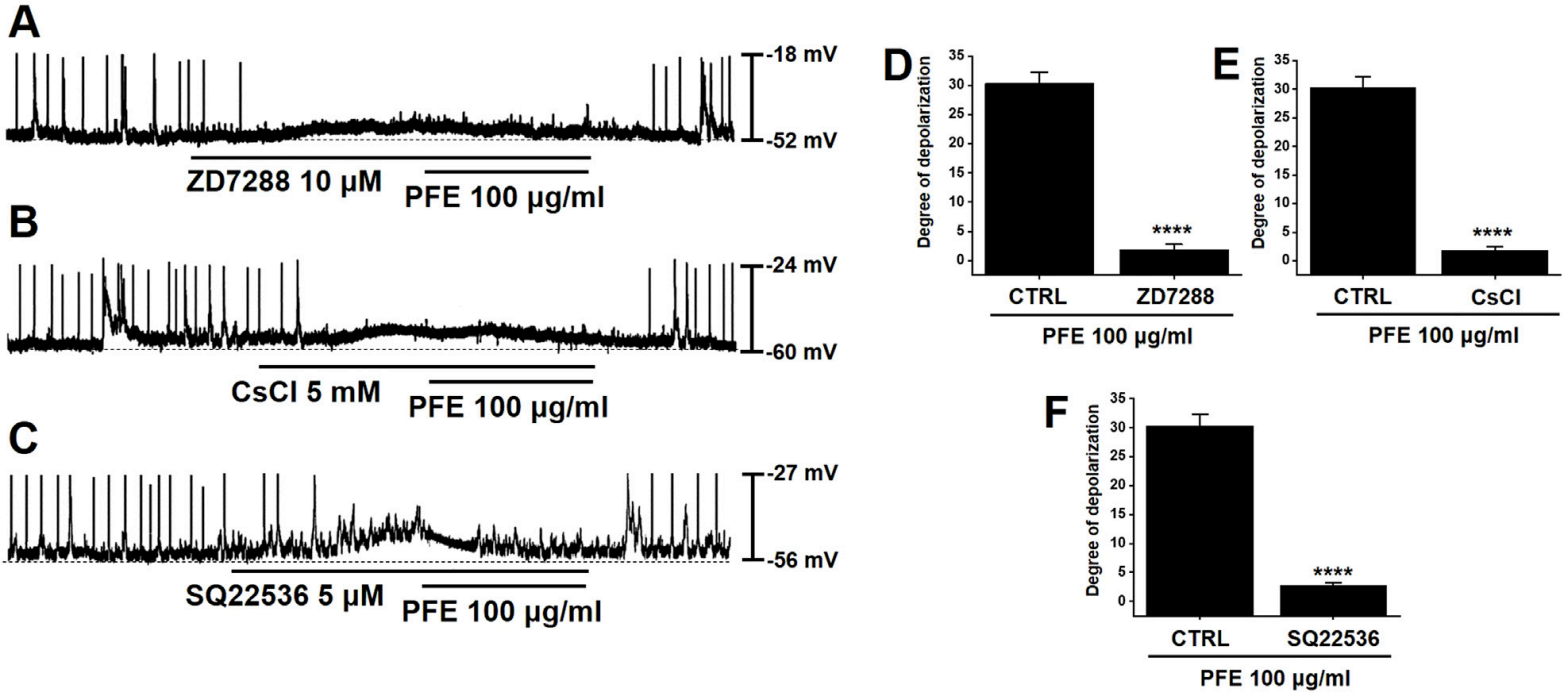
Inhibitory effects of HCN channel blockers and adenylate cyclase inhibition on PFE-induced modulation of pacemaker potentials in ICCs from the murine colon. **(A,B)** Application of ZD7288 (10 μM) and CsCl (5 mM), both known inhibitors of HCN channels, suppressed spontaneous pacemaker potentials and abolished PFE-induced responses in colonic ICCs. **(C)** SQ22536 (5 μM), an inhibitor of adenylate cyclase, also attenuated pacemaker activity and prevented the effects of PFE. **(D–F)** Quantitative summary of changes in pacemaker potential depolarization following PFE treatment. Bars indicate mean ± SE. ****p < 0.0001 compared to control. *CTRL: Control.*

Previous studies have indicated that HCN channels contribute to the generation of pacemaker activity in large intestinal ICCs (Choi et al., 2022). To investigate the involvement of HCN channels in the PFE-induced modulation of pacemaker activity, specific HCN channel inhibitors were applied. Treatment with ZD7288 or CsCl, both known HCN blockers, inhibited spontaneous pacemaker potentials in large intestinal ICCs and abolished the effects of PFE on these potentials (n = 7, Figures 3A,B). Given the regulatory role of intracellular cAMP on HCN channels, we also tested SQ22536, an adenylate cyclase inhibitor. Application of SQ22536 similarly suppressed pacemaker activity and prevented PFE-induced responses (n = 7, Figure 3C). The overall impact of HCN channel inhibition and cAMP blockade on PFE-induced effects is summarized in Figures 3D–F. These findings indicate that HCN channels are essential not only for basal pacemaker activity in large intestinal ICCs but also for mediating the effects of PFE.

### 3.4 Identification of muscarinic receptor subtypes targeted by PFE in in murine large intestinal ICCs

**FIGURE 4:**
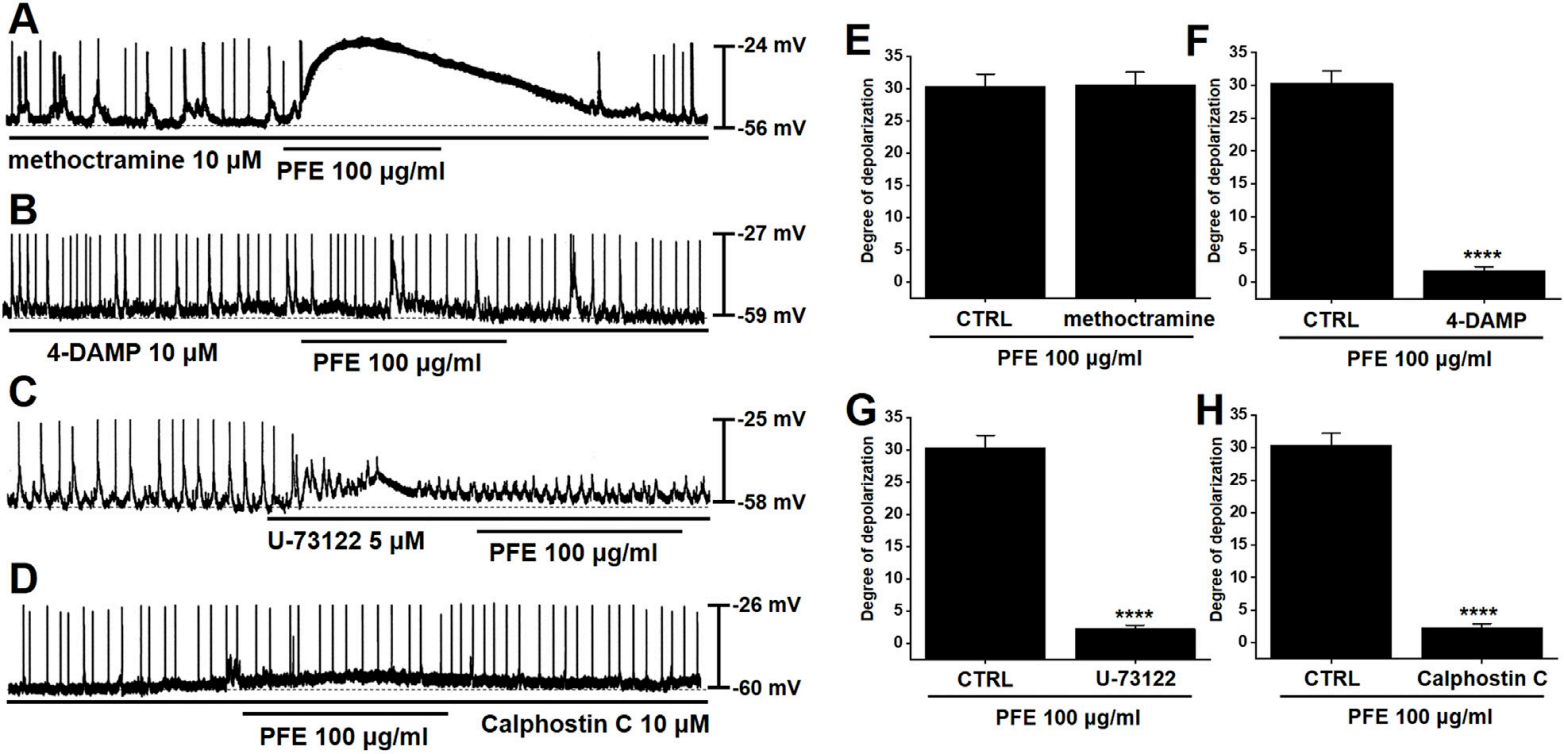
Involvement of muscarinic receptor subtypes and PLC/PKC signaling pathways in PFE-induced depolarizations in ICCs from the murine colon. **(A)** PFE continued to induce depolarization of pacemaker potentials in the presence of methoctramine, a selective M2 receptor antagonist. **(B)** However, pretreatment with 4-DAMP, a selective M3 receptor antagonist, abolished the depolarizing effect of PFE. **(C,D)** The depolarizing action of PFE was also blocked by U-73122 (5 μM), a PLC inhibitor, and by calphostin C (10 μM), a PKC inhibitor. **(E–H)** Bar graphs summarize the inhibitory effects of receptor antagonists and pathway inhibitors on PFE-induced depolarizations. Bars indicate mean ± SE. ****p < 0.0001 compared to control. *CTRL: Control.*

Muscarinic receptor activation is known to depolarize GI smooth muscle membranes (Ehlert et al., 2012), and previous studies have reported that M2 and M3 muscarinic receptor subtypes are predominantly expressed in GI ICCs (Drumm et al., 2020). To identify which receptor subtypes mediate the depolarizing effects of PFE, we employed selective muscarinic receptor antagonists. ICCs were treated with either methoctramine (10 μM), a selective M2 receptor antagonist, or 4-DAMP (10 μM), a selective M3 receptor antagonist. Neither antagonist alone altered the pacemaker potentials; furthermore, methoctramine pretreatment did not affect PFE-induced depolarizations (Figure 4A). In contrast, pretreatment with 4-DAMP completely blocked PFE-induced depolarization responses (Figure 4B). To further assess the signaling pathway involved, we tested the effect of U73122, a phospholipase C (PLC) inhibitor. Application of U73122 (5 μM) suppressed pacemaker potentials and abolished PFE-induced depolarizations (Figure 4C). Additionally, the involvement of protein kinase C (PKC) was examined using calphostin C (10 μM), a specific PKC inhibitor. While calphostin C alone had no effect on pacemaker activity, it prevented the PFE-induced depolarizing effect (Figure 4D). The summarized results of muscarinic receptor subtype inhibition and PLC/PKC pathway blockade on PFE-induced responses are shown in Figures 4E–H. Collectively, these findings suggest that PFE modulates pacemaker potentials in ICCs primarily via M3 muscarinic receptors, and that the PLC-PKC signaling cascade may be involved in mediating these effects.

### 3.5 Effects of mitogen-activated protein kinases (MAPKs) on PFE-induced responses in murine large intestinal ICCs

**FIGURE 5:**
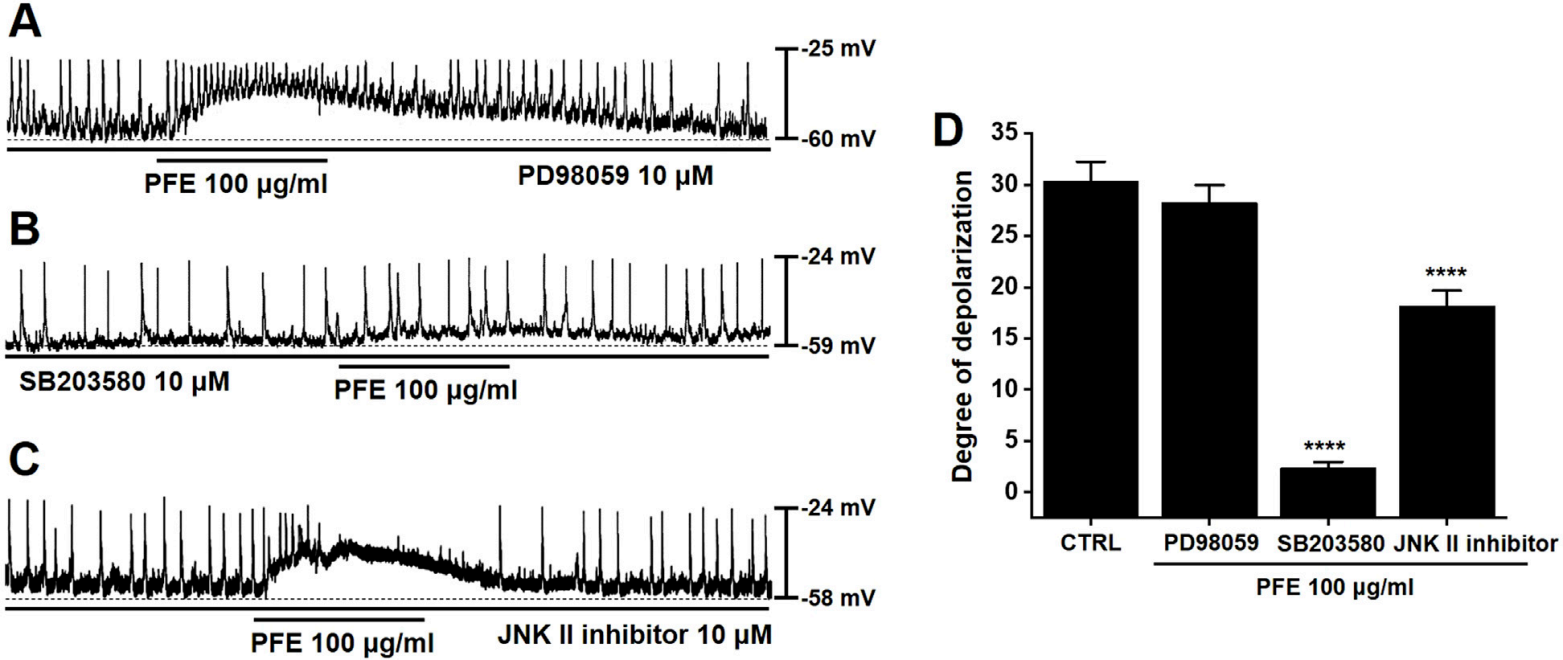
Influence of tyrosine kinase and MAPK pathway inhibition on PFE-induced depolarizations in ICCs from the murine colon. **(A)** PD98059 (10 μM), a selective inhibitor of ERK1/2 (p42/44 MAPK), had no significant impact on PFE-induced modulation of pacemaker activity. **(B)** In contrast, SB203580 (10 μM), a p38 MAPK inhibitor, suppressed the depolarizing effects of PFE. **(C)** Similarly, inhibition of JNK signaling with a JNK-II inhibitor (10 μM) abolished the PFE-induced depolarizations. **(D)** Graphical summary of the effects of MAPK pathway inhibitors on PFE-mediated changes in pacemaker potential frequency. Bars indicate mean ± SE. ****p < 0.0001 compared to control. *CTRL: Control.*

To evaluate the involvement of MAPKs in PFE-induced responses in large intestinal ICCs, specific MAPK pathway inhibitors were employed. Application of PD98059, a selective inhibitor of p42/44 MAPK (ERK1/2), had no significant effect on PFE-induced depolarizations in large intestinal ICCs (n = 6, Figure 5A). In contrast, pretreatment with SB203580, a p38 MAPK inhibitor, effectively suppressed the effects of PFE (n = 8, Figure 5B). Additionally, inhibition of JNK signaling using a JNK-II specific inhibitor also blocked PFE-induced depolarizations (n = 7, Figure 5C). A summary of the effects of these MAPK inhibitors on PFE responses is presented in Figure 5D. These findings suggest that p38 MAPK and JNK pathways, but not ERK1/2, are involved in mediating the depolarizing actions of PFE in large intestinal ICCs.

### 3.6 Effects of PFE on zymosan-induced colonic changes

**FIGURE 6:**
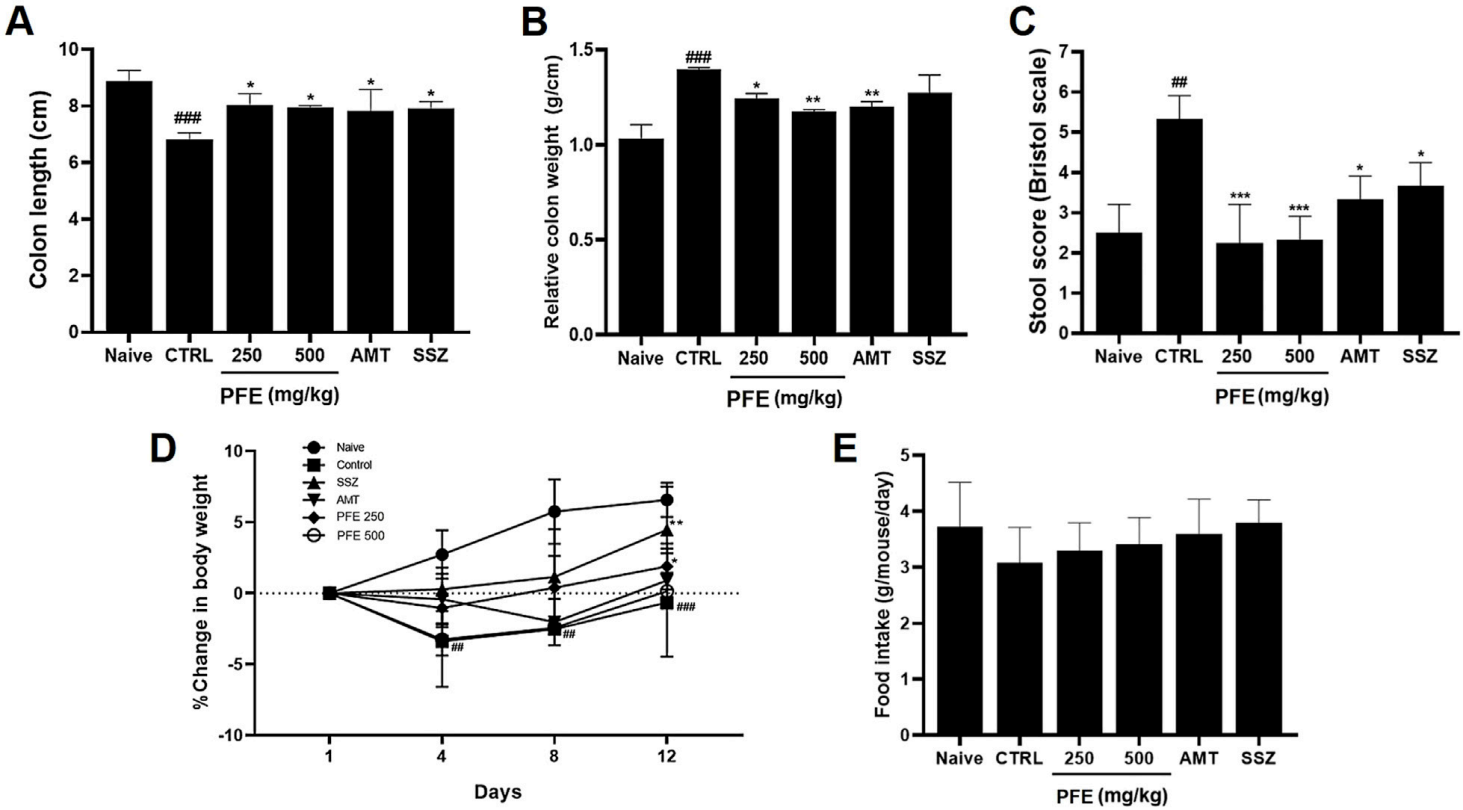
Influence of PFE on colonic parameters, stool consistency, body weight, and food consumption in a zymosan-induced IBS model. **(A)** The length of the colon, **(B)** the weight of the colon, and **(C)** the stool consistency scores were assessed in a zymosan-induced model of IBS. Furthermore, the effects of PFE on **(D)** body weight and **(E)** food intake were measured over the experimental period. Bars indicate mean ± SE. #p < 0.05, ##p < 0.01, and ###p < 0.001 compared to naïve controls; *p < 0.05, **p < 0.01, and ***p < 0.001 compared to control group. *CTRL: Control.*

We conducted experiments using a zymosan-induced IBS animal model to assess the effects of PFE. After PFE administration, we evaluated colon length, colon weight, and stool consistency. The colon length of zymosan-induced mice was significantly reduced compared to naïve mice; however, PFE treatment restored it to near-normal levels [8.89 ± 0.36 cm in naïve, 6.82 ± 0.23 cm in control (###p < 0.001), 8.04 ± 0.40 cm at 250 mg/kg PFE (*p < 0.05), 7.96 ± 0.06 cm at 500 mg/kg PFE (*p < 0.05), 7.83 ± 0.77 cm at AMT (*p < 0.05), and 7.93 ± 0.23 cm at SSZ (*p < 0.05); Figure 6A]. Additionally, colon weight, which significantly increased in the zymosan-induced group, was reduced upon PFE administration [1.03 ± 0.08 g in naïve, 1.39 ± 0.01 g in control (###p < 0.001), 1.24 ± 0.03 g at 250 mg/kg PFE (*p < 0.05), 1.18 ± 0.01 g at 500 mg/kg PFE (**p < 0.01), 1.20 ± 0.03 g at AMT (**p < 0.01), and 1.27 ± 0.10 g at SSZ; Figure 6B]. Furthermore, stool consistency, which was altered by zymosan, improved following PFE treatment [2.50 ± 0.71 in naïve, 5.33 ± 0.58 in control (##p < 0.01), 2.25 ± 0.96 at 250 mg/kg PFE (***p < 0.001), 2.33 ± 0.58 at 500 mg/kg PFE (***p < 0.001), 3.33 ± 0.58 at AMT (*p < 0.05), and 3.67 ± 0.58 at SSZ (*p < 0.05); Figure 6C]. Moreover, PFE administration reversed the weight loss induced by zymosan (Figure 6D), while food intake remained unchanged across groups (Figure 6E). These results indicate that PFE effectively alleviated colonic dysfunction, mitigated weight loss, and did not influence food consumption in a zymosan-induced IBS mouse model.

### 3.7 Effect of PFE on colon tissue, TNF-α levels and pain-associated behaviors

**FIGURE 7:**
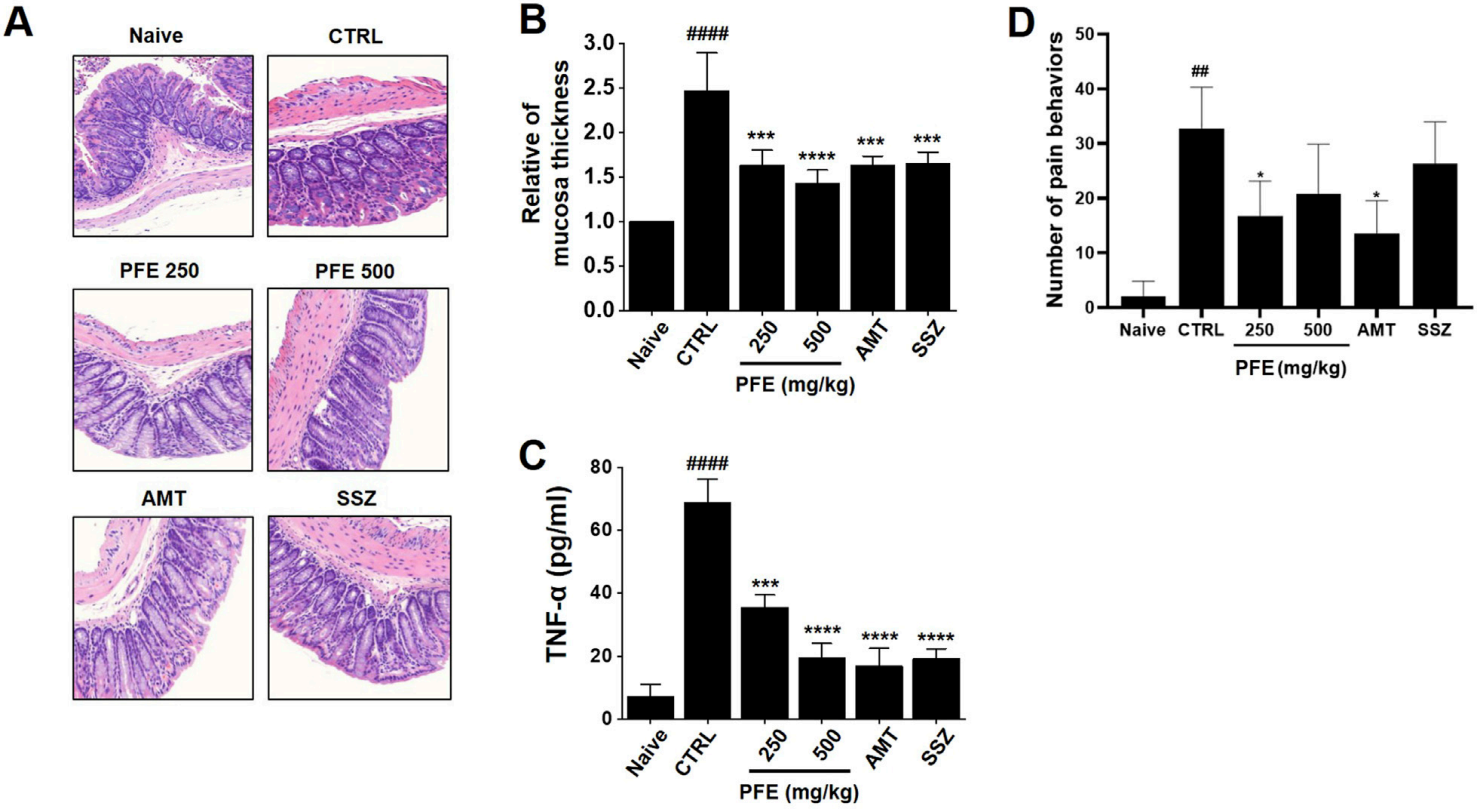
Impact of PFE on histological changes, TNF-α expression, and pain-related behavior in a colonic inflammation model. **(A)** H&E staining was conducted, with structural changes in the colon observed at ×50 magnification. **(B)** The thickness of the colonic mucosa was quantitatively measured to assess tissue integrity. **(C)** The expression levels of tumor necrosis factor-alpha (TNF-α), a key inflammatory marker, were analyzed using real-time quantitative PCR (RT-qPCR) to determine the inflammatory response following PFE treatment. **(D)** Pain-related behaviors were assessed to examine the potential analgesic effects of PFE in the experimental model. Bars indicate mean ± SE. ##p < 0.01, and ####p < 0.0001 vs. naïve. *p < 0.05, ***p < 0.001, ****p < 0.0001 vs. control. *CTRL: Control. TNF-*α*: Tumor Necrosis Factor-α.*

H&E staining revealed significant histological alterations in the colon tissues of zymosan-treated control mice, showing increased tissue thickness compared to normal mice. However, following treatment with PFE, the tissue thickness was restored to levels similar to those of the normal control group (Figures 7A,B). Colon samples were collected for analysis on day 4 post-colitis induction with zymosan. The control group displayed a significant increase in TNF-α expression, reflecting elevated levels associated with inflammation. In contrast, treatment with 250 and 500 mg/kg PFE, as well as the positive control group, led to a significant reduction in TNF-α levels (Figure 7C). Furthermore, pain-related behaviors were notably elevated on day 4, but PFE treatment significantly decreased these behaviors, which are indicative of visceral pain (Figure 7D). These results demonstrate that PFE effectively improved colonic tissue integrity, reduced TNF-α expression, and mitigated pain-related symptoms in the zymosan-induced IBS mouse model.

### 3.8 Effect of PFE on fecal microbial composition

**FIGURE 8:**
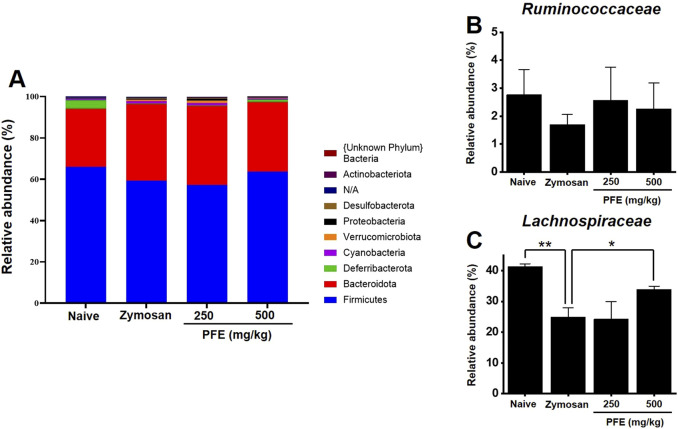
Composition of fecal microbiota at the phylum and family levels. **(A)** Bar chart illustrating the relative abundance of bacterial phyla across experimental groups. **(B,C)** Comparative analysis of the relative abundance of the Ruminococcaceae and Lachnospiraceae families, respectively, in each group. Bars indicate mean ± SE. *p < 0.05. **p < 0.01.

To assess the impact of PFE on the composition of the gut microbiota, fecal samples from each experimental group were analyzed. Differences in microbial communities were detected at the phylum level among the groups (Figure 8A). At the family level, no notable change was observed in the abundance of *Ruminococcaceae* (Figure 8B). In contrast, *Lachnospiraceae* levels were elevated following PFE treatment (Figure 8C).

### 3.9 Effects of PFE on TRP channel currents

**FIGURE 9:**
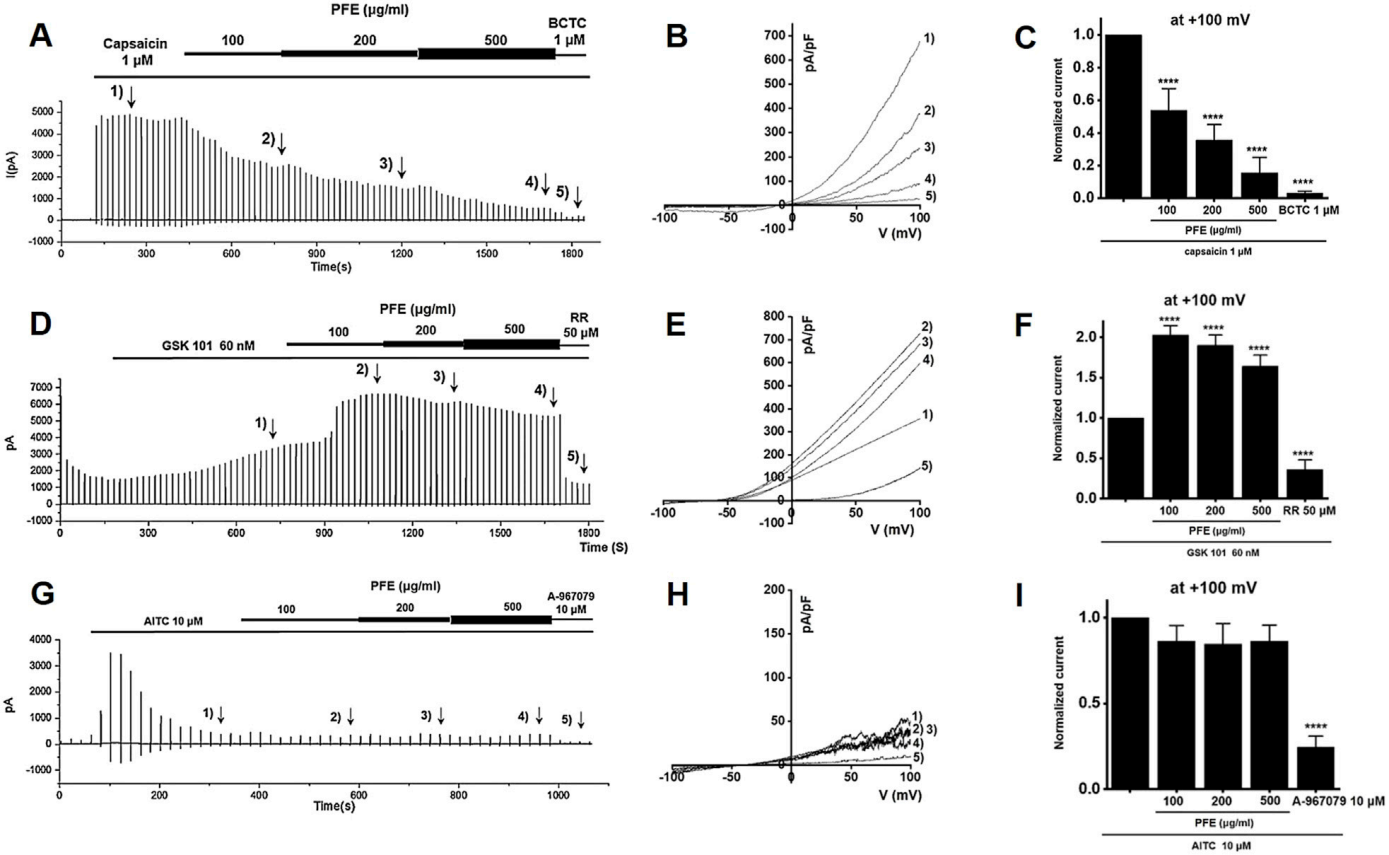
Modulatory effects of PFE on TRP channel currents (TRPV1, TRPV4, and TRPA1). Representative whole-cell patch-clamp recordings illustrate the effects of PFE at concentrations of 100, 200, and 500 μg/mL on **(A)** TRPV1, **(D)** TRPV4, and **(G)** TRPA1 currents. Capsaicin (TRPV1), GSK101 (TRPV4), and AITC (TRPA1) were used as respective agonists to activate each channel. Specificity of the recorded currents was confirmed using selective antagonists: BCTC (TRPV1), ruthenium red (TRPV4), and A-967079 (TRPA1). **(B,E,H)** I–V relationships demonstrate the effects of varying PFE concentrations on each TRP channel’s current–voltage characteristics. **(C,F,I)** Statistical comparisons of the relative changes in TRP currents reveal that PFE inhibited TRPV1 activity, enhanced TRPV4 activity, and had no effects on TRPA1 currents. Bars indicate mean ± SE. ****p < 0.0001 vs. control.

To examine how PFE influences TRP channels, whole-cell patch-clamp recordings were performed using HEK293T cells transfected with TRPV1, TRPV4, or TRPA1. For TRPV1, current–voltage (I–V) curves were obtained using a ramp protocol from −100 mV to +100 mV. Capsaicin was applied to activate TRPV1 currents (Abdel-Salam and Mózsik, 2023), and BCTC was used to confirm TRPV1 specificity (Heber et al., 2020). PFE treatment significantly suppressed TRPV1 currents at concentrations of 100, 200, and 500 μg/mL (****p < 0.0001), as shown in Figures 9A–C. In the case of TRPV4, GSK101 was used to induce TRPV4 currents (Baratchi et al., 2019), and ruthenium red (RR) served as a selective blocker (Cho et al., 2014). Under these conditions, PFE significantly enhanced TRPV4-mediated currents at all tested concentrations (****p < 0.0001; Figures 9D–F). For TRPA1, cells were activated using AITC (Ohashi et al., 2023), and A967079 was applied to confirm channel specificity (Gyamfi et al., 2020). In contrast to its effects on TRPV channels, PFE did not alter TRPA1-mediated currents at any concentration tested (Figures 9G–I). These findings indicate that PFE modulates TRPV1 and TRPV4 channel activity, while having no significant impact on TRPA1, suggesting a specific involvement of TRPV1 in the mechanism underlying PFE-mediated relief of visceral pain hypersensitivity.

### 3.10 Effects of PFE on NaV 1.5 and 1.7 currents

**FIGURE 10:**
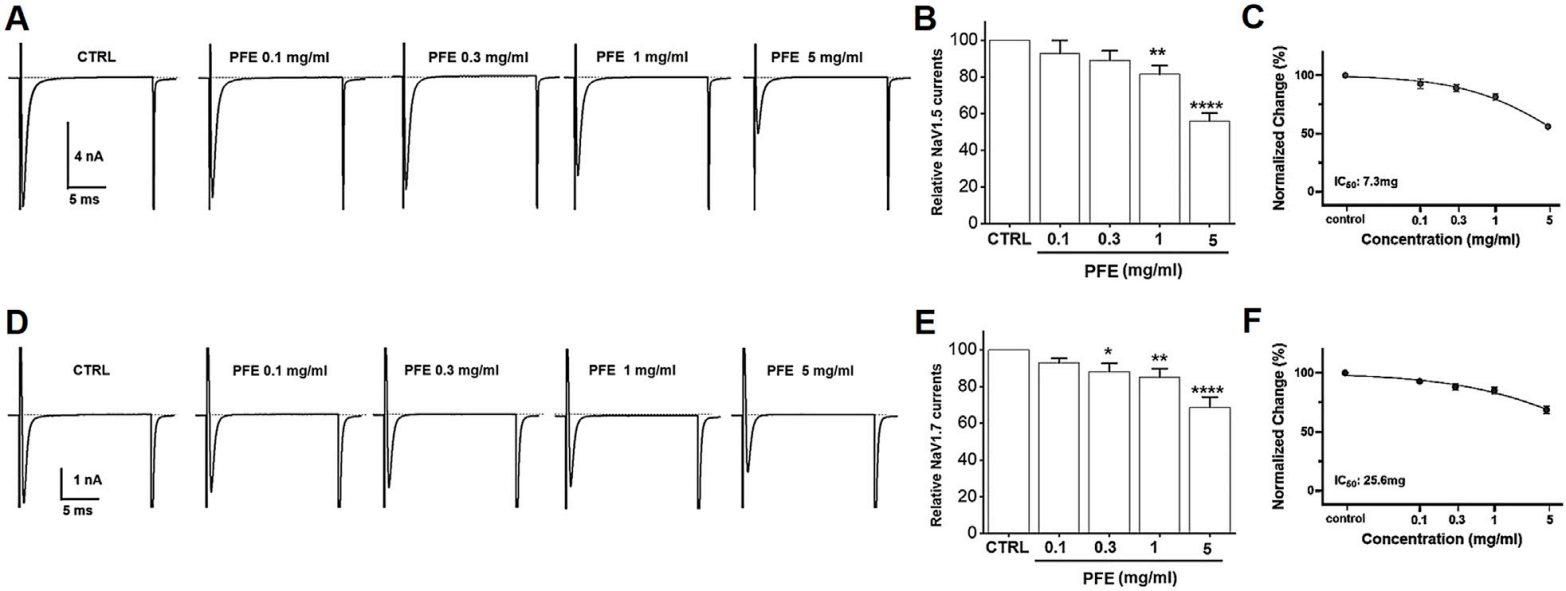
Modulatory effects of PFE on NaV1.5 and NaV1.7 currents in HEK293T cells. Representative whole-cell patch-clamp recordings showing the dose-dependent effects of PFE at concentrations of 0.1, 0.3, 1, and 5 mg/mL on **(A)** NaV1.5 and **(D)** NaV1.7 currents. **(B–E)** Statistical summaries depict the relative changes in current amplitudes for NaV1.5 and NaV1.7 channels following PFE treatment, illustrating the extent of inhibition across concentrations. **(C–F)** Normalized current analyses reveal the potency of PFE in modulating NaV, with estimated IC_50_ values of 7.3 mg/mL for NaV1.5 and 25.6 mg/mL for NaV1.7, indicating greater sensitivity of NaV1.5 to PFE. Bars indicate mean ± SE. *p < 0.05, **p < 0.01, and ****p < 0.0001 vs. control. *CTRL: Control.*

PFE markedly inhibited NaV1.5 currents in a concentration-dependent manner, reducing peak inward currents by 92.8% ± 7.1% at 0.1 mg/mL, 89.1% ± 5.2% at 0.3 mg/mL, 81.4% ± 4.7% at 1 mg/mL (**p < 0.01), and 55.9% ± 4.4% at 5 mg/mL (****p < 0.0001), with an estimated IC_50_ of 7.3 mg/mL (Figures 10A–C). Similarly, NaV1.7 currents were suppressed by 92.9% ± 2.4% at 0.1 mg/mL, 88.2% ± 4.5% at 0.3 mg/mL (*p < 0.05), 85.0% ± 4.7% at 1 mg/mL (**p < 0.01), and 68.7% ± 5.4% at 5 mg/mL (****p < 0.0001), with an IC_50_ of 25.6 mg/mL (Figures 10D–F). These findings imply that NaV1.5 and NaV1.7 channels are key targets involved in GI regulation and the antinociceptive effects of PFE.

## 4 Discussion

In this study, we investigated the therapeutic potential of the PFE in a zymosan-induced colitis mouse model, which mimics key features of IBS, including inflammation, visceral hypersensitivity, and motility dysfunction. Our findings demonstrate that PFE modulates ICCs pacemaker activity, alleviates colonic inflammation, and reduces visceral pain, indicating its potential as a therapeutic candidate for IBS management.

PF, a traditional medicinal botanical drug, has long been utilized in East Asian medicine for the treatment of various GI disorders (Jang et al., 2018). It has been reported to possess anti-inflammatory, prokinetic, and analgesic properties, making it a promising candidate for digestive health applications (Shin et al., 2006; Kim B. J. et al., 2013; Jang et al., 2018; Khan et al., 2019; Kim et al., 2019). Previous studies have shown that PF extracts can enhance GI motility, regulate smooth muscle contraction, and exert protective effects against colitis and gastric ulcers (Shin et al., 2006; Lee et al., 2009; Kim B. J. et al., 2013; Kim K. S. et al., 2013; Kang and Kim, 2016). The bioactive metabolites in PF, such as naringin and poncirin, have been implicated in modulating gut motility and reducing inflammation, further supporting its potential therapeutic role in IBS and related disorders (Kim et al., 2016; Cao et al., 2018; Lee K et al., 2024; Wu et al., 2024). Additionally, various natural products, including botanical medicines, have been widely studied for their efficacy in treating IBS and other GI conditions (Zhang et al., 2024).

Traditional remedies containing bioactive plant-derived metabolites, such as PF, ginger, peppermint, and licorice, have demonstrated beneficial effects in alleviating gut inflammation, modulating intestinal motility, and reducing visceral pain (Marasco et al., 2025). These natural treatments offer promising alternatives or adjuncts to conventional IBS therapies due to their multifaceted mechanisms of action and favorable safety profiles.

IBS is a prevalent and chronic GI disorder that significantly impacts patients’ quality of life (Marasco et al., 2025; Nozu et al., 2025). Despite its high prevalence, the pathophysiology of IBS remains incompletely understood, and effective treatment options are limited (Zhang et al., 2024; Jeong et al., 2025). The complex interplay between gut motility dysfunction, visceral hypersensitivity, immune activation, and gut microbiota dysbiosis necessitates ongoing research to develop targeted therapies (Zhang et al., 2024; Jeong et al., 2025). Given the heterogeneity of IBS symptoms and its substantial burden on healthcare systems worldwide, the exploration of novel therapeutic approaches, such as natural metabolites with anti-inflammatory, prokinetic, and analgesic properties, is of paramount importance. Research into PFE and other botanical extracts may pave the way for safer, more effective treatments that address the multifaceted nature of IBS.

One of the primary findings of this study is that PFE significantly influenced ICCs pacemaker activity (Figure 2). ICCs are essential for regulating smooth muscle activity and maintaining normal GI motility (Lee J et al., 2024; Baker et al., 2025). Dysregulation of ICCs has been implicated in IBS pathophysiology, contributing to both delayed and accelerated GI transit times (Foong et al., 2020; Patejdl, 2024). The ability of PFE to modulate ICCs function suggests that it may help restore normal colonic motility, thereby improving symptoms associated with IBS. In addition, electrophysiological investigations demonstrated that PFE-induced pacemaker modulation was mediated by HCN channels, as blockade with ZD7288, CsCl, or SQ22536 abolished the effects of PFE (Figure 3). Furthermore, PFE was found to act through M3 muscarinic receptors, with 4-DAMP and downstream PLC-PKC signaling inhibitors effectively blocking its depolarizing action in ICCs (Figure 4). Additionally, MAPK signaling was involved in this response, as inhibition of p38 MAPK or JNK, but not ERK1/2, suppressed PFE-induced effects (Figure 5). These findings suggest a complex but specific intracellular signaling cascade underlying PFE’s prokinetic actions on ICCs.

Furthermore, PFE effectively mitigated colonic dysfunction induced by zymosan, as indicated by the restoration of colon length and reduction in colon weight (Figure 6). These findings suggest that PFE may counteract the inflammatory and structural changes associated with colitis. Histological analysis further confirmed the protective effects of PFE, as it significantly preserved tissue integrity and reduced signs of inflammation, such as epithelial damage and thickening of colonic tissues (Figures 7A,B). Importantly, PFE administration significantly downregulated TNF-α expression, a key pro-inflammatory cytokine involved in IBS pathogenesis (Figure 7C). Elevated TNF-α levels have been associated with increased gut permeability, immune activation, and heightened pain sensitivity in IBS patients. The observed reduction in TNF-α expression following PFE treatment underscores its anti-inflammatory potential in IBS-like conditions.

Pain-related behaviors, including abdominal licking, stretching, and pressing, were significantly elevated in the zymosan-induced colitis model, reflecting the heightened visceral pain characteristic of IBS. PFE administration effectively reduced these behaviors, suggesting its analgesic potential in managing IBS-associated pain (Figure 7D). The underlying mechanism of PFE’s pain-relieving effects was further explored through electrophysiological studies, which revealed that PFE significantly modulated pain-related ion channels, particularly TRP and NaV channels.

TRPV1, TRPV4, and TRPA1 channels are well-established mediators of visceral pain hypersensitivity in IBS. Our findings indicate that PFE significantly inhibited TRPV1 channel activity (Figures 9A–C) and enhanced TRPV4 currents (Figures 9D–F), whereas it showed no significant modulatory effect on TRPA1 channels (Figures 9G–I). Given that TRPV1 is known to contribute to inflammatory pain via activation by noxious stimuli, its suppression by PFE likely played a role in reducing visceral pain sensitivity. Conversely, TRPV4 and TRPA1 do not seem to be involved in IBS regulation by PFE. Among these, I would like to mention TRPV4. Our results indeed demonstrated that PFE enhanced TRPV4 currents. However, previous studies have suggested that inhibition of TRPV4, rather than its activation, is generally associated with the improvement of IBS-related symptoms such as visceral hypersensitivity. Therefore, although PFE increased TRPV4 currents *in vitro*, this effect is unlikely to contribute to its therapeutic action in IBS. Instead, our data strongly indicate that the beneficial effects of PFE on IBS are primarily mediated through modulation of TRPV1 activity, which is consistent with its known role in visceral pain regulation. Therefore, we concluded that TRPV4 activation by PFE is an off-target phenomenon, while TRPV1 serves as the key channel involved in the therapeutic mechanism of PFE in IBS. Additionally, PFE significantly inhibited NaV1.5 and NaV1.7 channel activity (Figure 10). NaV1.5 is primarily involved in GI motility, and its dysregulation has been linked to gut dysmotility disorders, including IBS (Strege et al., 2003; Verstraelen et al., 2015). In contrast, NaV1.7 plays a critical role in pain perception by amplifying nociceptive signals (Jiang et al., 2021). The observed suppression of NaV1.7 currents by PFE suggests that it may exert analgesic effects by reducing hyperexcitability of nociceptive neurons. The dose-dependent inhibitory effects of PFE on these ion channels further support its potential role in alleviating IBS-associated visceral pain. Future research should also investigate the involvement of other ion channels, such as calcium-activated potassium channels and chloride channels, in the pathophysiology of IBS and the potential modulatory effects of PFE on these targets (Lin and Yu, 2018; Fan et al., 2021). Expanding our understanding of additional ion channel interactions may provide further insights into the comprehensive mechanisms underlying PFE’s therapeutic effects.

In addition to these physiological effects, we also examined the impact of PFE on gut microbiota composition. At the phylum level, microbial communities in the zymosan-induced IBS group differed from those in the naïve and PFE-treated groups (Figure 8A). Although an increase in *Firmicutes* is commonly reported in IBS (Duan et al., 2019), our study observed a decreasing trend. A similar reduction in *Firmicutes* has also been noted in previous studies using gintonin (Choi et al., 2024b), suggesting that this may be a characteristic response in zymosan-induced models. Fecal microbiota analysis further revealed that while the abundance of *Ruminococcaceae* remained relatively unchanged, *Lachnospiraceae* levels were significantly increased following PFE administration (Figures 8B,C). Members of the *Lachnospiraceae* family contribute to gut health, particularly by supporting the integrity of the intestinal barrier and modulation of inflammatory responses (Ćesić et al., 2023; Zaplana et al., 2024). The observed increase in this bacterial family suggests that PFE may help restore microbial balance and contribute to its overall therapeutic efficacy in IBS. However, the microbiota results showed some variability depending on the experimental conditions. Maintaining consistent animal states throughout the study was challenging, and external factors such as stress during drug administration may have influenced microbial composition. In certain cases, differences between groups were minimal. Therefore, more rigorously controlled conditions will be required in future studies to ensure reproducibility and accuracy in microbiota-related findings. Moreover, this study was limited to a diarrhea-predominant IBS model induced by zymosan. To fully evaluate the therapeutic potential of PFE, future studies should also investigate its effects in other IBS subtypes, including constipation-predominant IBS. Additionally, only two concentrations of PFE (250 and 500 mg/kg) were tested; thus, future work should explore a wider dose range to better characterize the dose-response relationship and optimize therapeutic outcomes.

While our study provides strong evidence for the therapeutic potential of PFE in IBS, certain limitations should be considered. First, although the zymosan-induced colitis model effectively replicates inflammation and motility dysfunction seen in IBS, it does not fully encompass all subtypes of IBS, such as IBS with predominant constipation (IBS-C) or diarrhea (IBS-D). We fully acknowledge that IBS is a heterogeneous disorder, with varying underlying mechanisms and clinical presentations across subtypes such as IBS-D, IBS-C, and mixed-type (IBS-M). In the present study, we utilized a zymosan-induced post-inflammatory mouse model, which is most closely associated with the post-infectious or diarrhea-predominant subtype of IBS. This model exhibits transient colonic inflammation, altered motility, and visceral hypersensitivity without permanent mucosal damage—features commonly observed in IBS-D and post-infectious IBS (PI-IBS) patients. Moreover, the observed effects of PFE—including its ability to reduce TNF-α levels, modulate colonic motility via ICCs regulation, and attenuate pain-related behaviors—are particularly relevant to IBS-D pathophysiology, which is often associated with immune activation, gut dysmotility, and enhanced visceral pain perception. Although our current model does not fully encompass all IBS subtypes, our findings lay the groundwork for exploring PFE in broader IBS contexts. In future studies, we plan to examine the effects of PFE in other validated models that reflect constipation-predominant or mixed-type IBS, and to investigate how specific active metabolites may differentially affect motility and pain pathways across subtypes. Second, we utilized PFE without isolating specific active metabolites. Although this reflects traditional usage, it limits mechanistic clarity and may affect reproducibility. In addition, the dose range used in the current study was relatively narrow, focusing on a single effective concentration. A systematic evaluation of multiple doses will be essential to clarify the dose–response relationship of PFE, optimize its therapeutic efficacy, and minimize potential adverse effects. Third, while we focused on key ion channels implicated in visceral pain (TRPV1, TRPV4, TRPA1, NaV1.5, and NaV1.7), other relevant targets such as acid-sensing ion channels (ASICs), purinergic receptors, or serotonergic pathways were not explored. In addition, the *in vivo* assessments were limited to a short-term experimental window; thus, the long-term efficacy, safety, and pharmacokinetics of PFE remain to be investigated. Finally, as this study was conducted entirely in animals and cell lines, further translational studies, including clinical trials, will be required to confirm the therapeutic potential of PFE in human IBS patients. Taken together, future work will focus on testing PFE in multiple IBS models (IBS-D, IBS-C, and IBS-M), performing dose-ranging studies, isolating its active metabolites, expanding the scope of molecular targets, and evaluating chronic administration and clinical applicability.

In summary, PF (also known as “Jisil” in Korean and “Zhishi” in Chinese) has a long history of use in traditional East Asian medicine for treating various GI symptoms, including abdominal pain, indigestion, bloating, constipation, and diarrhea. It is commonly included in botanical formulas designed to regulate Qi, improve GI motility, and relieve food stagnation. Our findings provide a mechanistic basis that supports these traditional uses. Specifically, the observed effects of PFE on colonic motility via modulation of ICCs, the reduction in visceral pain through inhibition of nociceptive ion channels such as TRPV1 and NaV1.5/1.7, and the attenuation of intestinal inflammation are all consistent with the GI indications of PF in traditional medicine. Furthermore, the increase in beneficial gut microbiota (e.g., Lachnospiraceae) after PFE treatment aligns with the traditional notion of restoring gut harmony and balance. By identifying specific molecular targets and signaling pathways involved in the therapeutic effects of PF, our study bridges traditional empirical knowledge with modern pharmacological evidence.

## 5 Conclusion

Taken together, these findings strongly support the conclusion that PFE exerts therapeutic effects in a zymosan-induced IBS model by modulating ICC activity, reducing colonic inflammation, alleviating visceral pain, and influencing pain-related ion channels. Furthermore, alterations in gut microbiota composition associated with PFE treatment, including a trend toward increased levels of beneficial bacterial families, may contribute to the maintenance of intestinal homeostasis. These findings suggest that PFE holds promise as a potential treatment option for IBS and related gastrointestinal disorders. Further clinical investigations will be necessary to confirm its efficacy and safety in human populations.

## Data Availability

The original data are available upon reasonable request to the corresponding author. The data are not publicly available due to privacy and ethical restrictions.
